# Hydrostatic pressure-generated reactive oxygen species induce osteoarthritic conditions in cartilage pellet cultures

**DOI:** 10.1038/s41598-018-34718-8

**Published:** 2018-11-19

**Authors:** Bernhard Rieder, Anna M. Weihs, Adelheid Weidinger, Dorota Szwarc, Sylvia Nürnberger, Heinz Redl, Dominik Rünzler, Carina Huber-Gries, Andreas H. Teuschl

**Affiliations:** 1 Department Life Science Engineering, University of Applied Sciences Technikum Wien, 1200 Vienna, Austria; 2 Ludwig Boltzmann Institute for Experimental and Clinical Traumatology, AUVA Research Center, 1200 Vienna, Austria; 3 Department of Orthopedics and Trauma-Surgery, Division of Trauma-Surgery, Medical University of Vienna, 1090 Vienna, Austria; 4 University Clinic of Dentistry, Medical University of Vienna, 1090 Vienna, Austria; 5 Austrian Cluster for Tissue Regeneration, 1200 Vienna, Austria

## Abstract

Osteoarthritis (OA) is one of the most common causes of disability and represents a major socio-economic burden. Despite intensive research, the molecular mechanisms responsible for the initiation and progression of OA remain inconclusive. In recent years experimental findings revealed elevated levels of reactive oxygen species (ROS) as a major factor contributing to the onset and progression of OA. Hence, we designed a hydrostatic pressure bioreactor system that is capable of stimulating cartilage cell cultures with elevated ROS levels. Increased ROS levels in the media did not only lead to an inhibition of glycosaminoglycans and collagen II formation but also to a reduction of already formed glycosaminoglycans and collagen II in chondrogenic mesenchymal stem cell pellet cultures. These effects were associated with the elevated activity of matrix metalloproteinases as well as the increased expression of several inflammatory cytokines. ROS activated different signaling pathways including PI3K/Akt and MAPK/ERK which are known to be involved in OA initiation and progression. Utilizing the presented bioreactor system, an OA *in vitro* model based on the generation of ROS was developed that enables the further investigation of ROS effects on cartilage degradation but can also be used as a versatile tool for anti-oxidative drug testing.

## Introduction

Osteoarthritis (OA) is the most common type of arthritis, affecting 25% of the adult population. It has been forecast that in the US alone, approximately 50 million people will suffer from OA by the year 2020^1,2^. This degenerative joint disease is predominantly observed in elderly people, which historically resulted in the hypothesis that OA is a simple “wear-and-tear” disease of articular cartilage^3,4^. It was believed that the loss of articular cartilage subsequently results in altered biomechanics combined with cellular changes which over time led to severe changes of the subchondral bone, synovium, menisci, ligaments, periarticular muscles and nerves^5^. This hypothesis is supported by results of *in vivo* models in which mechanical instability of the knee joint was induced, e.g. by transection of the anterior cruciate ligament^6,7^ to promote excessive wear of cartilage structures.

Lately, OA has increasingly become regarded as an inflammation process causing an imbalance in the homeostasis of articular chondrocytes, ultimately resulting in progressive loss and destruction of articular cartilage. Similar to rheumatoid arthritis (RA), OA is associated with synovial inflammation but generally to a lesser extent (lower number of synovial fluid leukocytes than in RA). In contrast, OA is characterized by high levels of a number of pro-inflammatory cytokines and chemokines which result in the production of extracellular matrix-degrading enzymes such as matrix metalloproteinases (MMPs) responsible for the loss of articular cartilage^5^.

Despite over 20 years of research, the molecular mechanisms responsible for OA initiation and progression remain poorly understood. Nevertheless, it is now well accepted that the pathogenesis of OA is much more complex than just a “wear-and-tear” and that mechanical factors in the form of excessive and abnormal joint loading play a crucial role.

In this regard, different *in vivo* and *in vitro* OA models have been established to decipher the roles of specific factors contributing to the disease.

Most *in vitro* OA models use cartilage explants, primary (osteoarthritic) chondrocytes or mesenchymal stem cells (MSC) differentiated into the chondrogenic lineage and can be grouped according to the trigger utilized in the initiation of the catabolic process. The majority of studies involve the use of either cytokine treatment alone (such as the addition of pro-inflammatory cytokines IL-1β or TNF-α) or in combination with physical stimulation, such as osmotic pressure, physical injury/deformation and mechanical loading regimes^8–10^. In this regard cyclic hydrostatic pressure has been shown to increase both the production of nitric oxide as well as proteoglycan synthesis^11^ and to change the cellular ultrastructure^12,13^ of IL-1β-treated osteoarthritic chondrocytes. These findings underline the importance of mechanical stimulation for the hemostasis of not only healthy but also osteoarthritic chondrocytes.

In the last decade a number of studies have demonstrated that reactive oxygen species (ROS) are involved in the initiation and progression of OA^14,15^. So far only a few studies use adequate and physiological *in vitro* models to simulate elevated ROS levels to generate an OA model. In select studies osteoarthritic chondrocytes are generated by application of H_2_O_2_^16–20^, which is based on *in vivo* production of H_2_O_2_ by neutrophils and macrophages or by chondrocytes themselves in inflamed knee joints. In this regard, chondrocytes have been shown to produce superoxide radicals by activation of NADPH oxidase (nicotinamide adenine dinucleotide phosphate oxidase)^21^, which can subsequently dismute into H_2_O_2_. Moreover, Regan *et al*. demonstrated that joint fluids of OA patients are characterized by significantly decreased extracellular superoxide dismutase (SOD) levels compared to samples from healthy patients^22,23^, indicating a crucial role of uncontrolled superoxide levels in the initiation of OA.

Here we demonstrate that the application of hydrostatic pressure (HP) by compressed air induced the production of elevated levels of superoxide and other ROS species (determined via electron paramagnetic resonance measurements), which subsequently hindered chondrogenic development of MSC pellet cultures by downregulating expression of cartilage-specific proteins, such as collagen type II and glycosaminoglycans, and upregulating expression of collagen type I, matrix metalloproteinases and inflammatory cytokines. Moreover, the analysis of crucial signaling pathways revealed that applied hydrostatic pressure caused an enhanced activation of the OA-associated pathways MAPK/ERK and PI3K/Akt.

In this study, to the best of our knowledge, we are the first to show that acellular superoxide formation induced by a custom-made hydrostatic pressure system generates a degenerative OA-like environment for chondrogenic MSC pellets.

## Materials and Methods

If not indicated otherwise, all chemicals and reagents were purchased from Sigma Aldrich (St. Louis, MO, USA) and were of analytical grade.

### Cell isolation and culture

Human adipose tissue derived stromal cells (hASCs) were kindly provided by the Ludwig Boltzmann Institute for Experimental and Clinical Traumatology in cooperation with Red Cross Blood Transfer Service of Upper Austria. Cell isolation was performed in accordance with the relevant guidelines and regulations as described in Wolbank *et al*.^24^ with authorization of the local ethics committee (Province of Upper Austria) and informed consent of the donor. Briefly, the stromal vascular fraction was obtained via several washing steps of lipoaspirate with phosphate-buffered saline (PBS) followed by enzymatic digestion of the tissue. Subsequently, the cellular fraction was seeded on plastic dishes, allowing for selection between plastic-adherent and non-adherent cells. The plastic-adherent hASCs were further cultivated and frozen, according to the laboratory-specific standard operating procedures (SOPs).

For expansion, hASCs were cultured in DMEM:F12 (Lonza, Basel, Switzerland) supplemented with 10% foetal bovine serum (FBS; GE Healthcare, Little Chalfont, United Kingdom), 100 U/mL penicillin, 100 µg/mL streptomycin (1% P/S; Lonza, Basel, Switzerland) and 5 ng/mL basic fibroblast growth factor (bFGF; PeproTech, Rocky Hill, NJ, USA). This medium will be further referred to as expansion medium (EM). Cells were expanded on standard cell culture dishes (STARLAB, Hamburg, Germany) in a humidified incubator at 37 °C and 5% CO_2_. To avoid premature differentiation, cells were subcultured at 80–85% confluence. When the desired cell concentration was reached, hASCs were transferred into round bottom 96-well plates (SPL Life Sciences, Korea) and spun down for 5 min at 300 × g to form cell pellets. Each well contained 2.5 × 10^5^ cells and pellets were fully formed within 3 days after centrifugation. Pellets were differentiated with DMEM (Lonza, Basel, Switzerland) supplemented with 2 mM L-glutamine (Lonza, Basel, Switzerland), 1% P/S (Lonza, Basel, Switzerland), 1 mM sodium pyruvate, 10 mM HEPES, 50 µg/mL proline, 1x Insulin-transferrin-sodium selenite (ITS + 3), 100 nM dexamethasone (DEX), 170 µM ascorbic acid (AA), 10 ng/mL transforming growth factor-β 3 (TGF-β3; PeproTech, Rocky Hill, NJ, USA) and 10 ng/mL bone morphogenetic protein 6 (BMP-6; PeproTech, Rocky Hill, NJ, USA) for a total of 42 days. This medium will be further referred to as differentiation medium (DM). A partial medium change was performed on day 2 followed by a total medium change every 3–4 days until the end of the experiment.

### Custom-made hydrostatic pressure system

**Figure 1:**
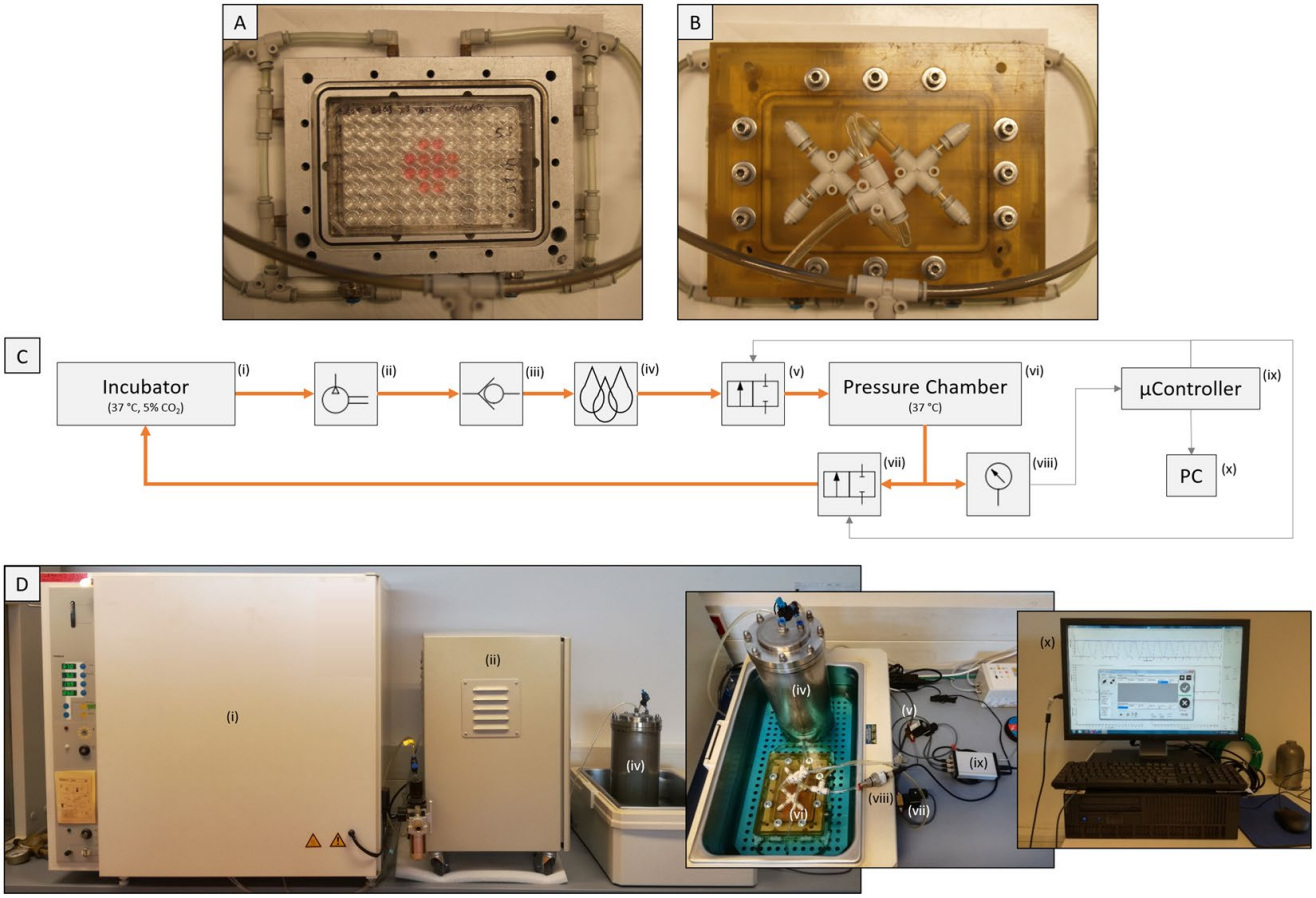
The hydrostatic pressure bioreactor system. (**A**) Aluminum baseplate of the pressure chamber with a total of 8 inlet ports (2 inlet ports on each side) for uniform air and pressure distribution within the chamber. The baseplate is housing the pellet culture (1 pellet per well filled with 250 µL of medium) in a 96 well plate. (**B**) The pressure chamber is closed with an ABS cover plate, which has a total of 6 outlet ports on top for fast and uniform air exhaustion. The cover plate is fastened with 12 stainless steel screws on the baseplate and an O-ring seal provides an airtight seal. (**C**) A schematic view of the custom-made bioreactor system: The incubator (i) provides CO_2_-buffered air which can be pressurized up to 8 bars in the compressor (ii) and is forwarded to the humidifier (iv). A back-pressure valve (iii) was integrated to allow only unidirectional flow to the humidifier. Then the moistened air is introduced into the pressure chamber (vi) and the maximum pressure value as well as the planned regime is controlled via the inlet (v) and outlet (vii) solenoid valves. The pressure chamber, which contains the stimulated pellets, can house any standard well plate format (96- to 6-well plates). The pressure in the chamber is measured with a pressure transducer (viii) and measured values are processed in the µController (ix) which controls the two solenoid valves according to the regime set by the user via the GUI/PC (x). (**D**) Assembled bioreactor system necessary to stimulate pellets. Humidifier and pressure chamber were placed in a waterbath to keep medium temperature constant at 37 °C.

The pressure chamber (Fig. 1A) consists of a milled aluminium baseplate which fits any type of multi-well plate ranging from 6 to 96 wells. The pressure is flushed into the inner chamber via inlets inside the walls of this baseplate. For uniform air and equal pressure distribution, each side is equipped with 2 inlet ports, which sums up to 8 inlet ports in total. The pressure chamber is closed with an acrylonitrile butadiene styrene (ABS) cover plate (Fig. 1B). The cover plate has 6 outlet ports on the top side which are regularly distributed to allow for uniform air exhaust. To provide an airtight seal, the pressure chamber has an O-ring seal between the two components of the chamber. The ABS cover plate is fastened with 12 stainless steel screws.

The pressure is regulated via two solenoid valves (Bürkert, Ingelfingen, Germany), one before the inlet ports and one after the inlet ports of the chamber (Fig. 1C). The valves are controlled by a microcontroller (Microchip Technology Inc., Chandler, AZ, USA), running a customized program coded in C. The user accesses the microcontroller via a graphical user interface (GUI) coded in C# with Visual Studio (Microsoft, Redmond, WA, USA). The program was specifically designed to enable the user to define the critical experiment parameters like pressure, cycle time, and total stimulation period. Furthermore, the GUI displays the pressure in real-time, which is measured by a pressure transducer (RS Components, Corby, UK) attached to the pressure chamber.

The pressure is generated by a commercially available air-cooled gas compressor (Jun-Air, Redditch, UK) (Fig. 1D) that draws in air from the incubator and compresses it to a maximum pressure of 8 bars. The air is moisturized in a custom-made humidifier to prevent evaporation of the medium in the wells inside the pressure chamber. After passing the pressure chamber, the air gets transferred back into the incubator, closing the loop.

### Experimental plan and mechanical stimulation protocol

**Figure 2:**
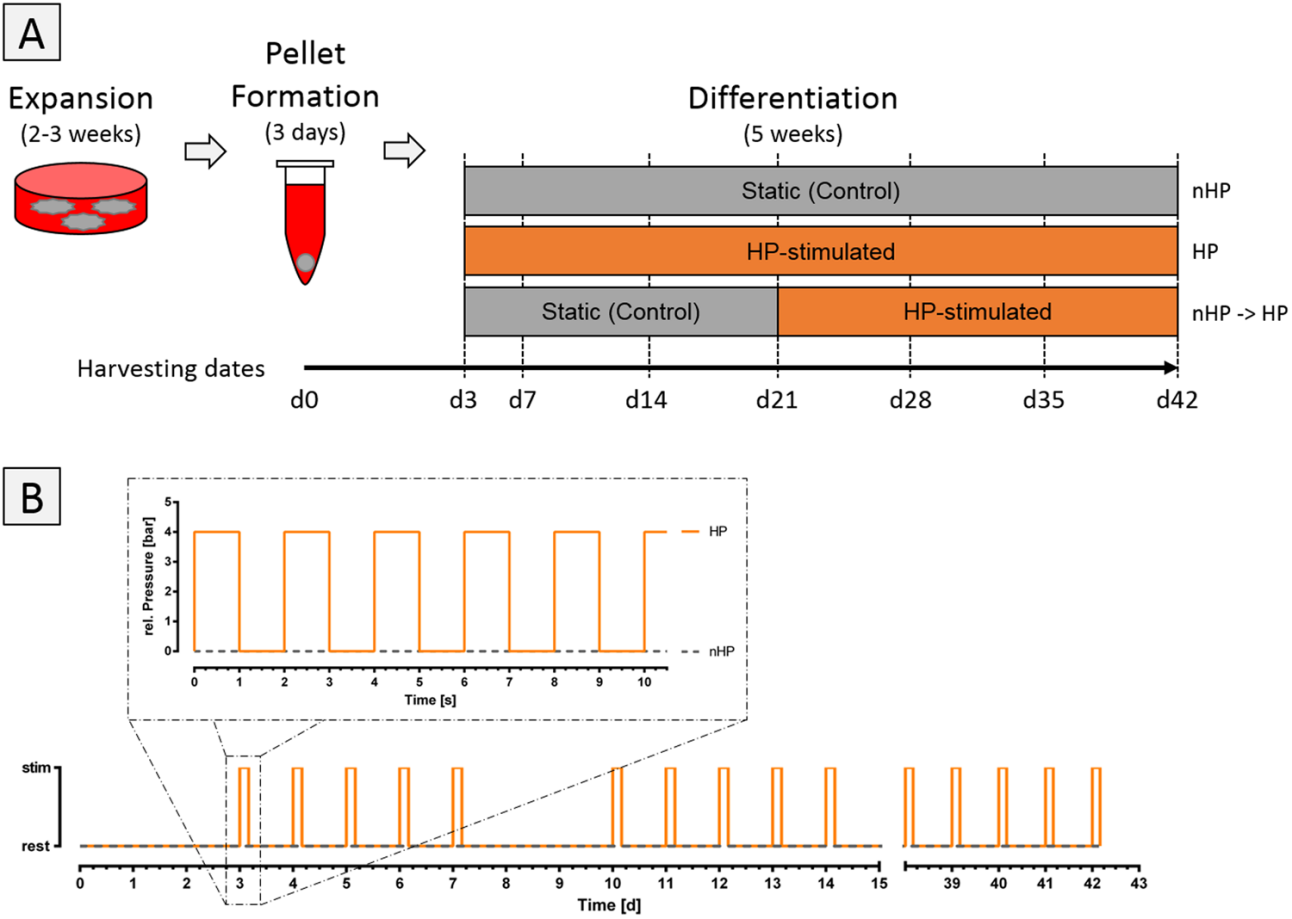
Experimental study set-up. (**A**) Human ASCs were cultured for 2–3 weeks until desired cell concentration was reached. Then, 250,000 cells per pellet were spun down to induce pellet formation, which was completed within 3 days. Pellets were divided into 3 groups: unstimulated static control for 42 days (nHP), hydrostatic pressure stimulated (HP) for 42 days, no stimulation for 21 days followed by HP stimulation for 21 days (nHP → HP). Pellets were harvested on day 0, 3 and 7 and from then on every seventh day for the total period of the experiment. (**B**) The hydrostatic pressure stimulation protocol. After 3 days of initial pellet formation, pellets were subjected to 4 hours of intermitted stimulation in a 2 s on/off manner with a maximum and minimum pressure of 4 and 0 bars, respectively. Each stimulation phase was followed by a no pressure period of 20 hours. This regime was repeated for 5 consecutive days per week with 2 resting days in-between. This pattern was repeated until the end of the experiment on day 42.

hASCs were cultured for 2–3 weeks until desired cell concentration was reached. Then, cells were harvested and spun down to form pellets. The day of harvesting and pelleting was defined as day 0 of the experiment. Pellets were divided into 3 experimental groups (no stimulation, HP stimulation, no stimulation for 21 days followed by 21 days of HP stimulation) (Fig. 2A) and cultured until day 42 with sample harvest every seven days. All experimental groups were subjected to DM for the complete period of the experiment.

HP-stimulated pellets were subjected to 4 hours of intermitted stimulation in a 2 s on/off manner with a maximum and minimum pressure of 4 and 0 bars, respectively. Each stimulation phase was followed by a no pressure period of 20 hours. This regime was repeated on 5 consecutive days per week. This pattern was repeated until the end of the experiment on day 42 (Fig. 2B).

### Histology and immunohistochemical (IHC) analysis

Harvested pellet cultures were fixed in 4% formaldehyde (Histofix®, Roth, Karlsruhe, Germany) for 24 hours at 4 °C and dehydrated with a graded series of ethanol steps (50% to 100%). Samples were embedded in paraffin, sectioned to 5 µm, and mounted on glass slides. To proceed with distinct stainings, sections were deparaffinized with xylene (Roth, Karlsruhe, Germany) and rehydrated with a graded series of ethanol washes to distilled water. Glycosaminoglycans (GAGs) were detected by alcian blue staining. Briefly, alcian blue stain was performed by Alcian blue 8GX (Sigma, St. Louis, MO, USA) for 30 min followed by rinsing with distilled water till the sections were clean. Then, slides were counterstained with Mayers Hemalum (Roth, Karlsruhe, Germany) for 2 min and rinsed in running tap water for 10 min. Afterwards, sections were dehydrated with a graded series of ethanol washes, cleared with xylene (Roth, Karlsruhe, Germany) and covered with a glass slide.

Collagen types I and II were detected via IHC staining in order to assess the quality of cartilage tissue formation of the pellet cultures. Briefly, collagen type I staining was performed by drying paraffin-coated samples at 40 °C overnight while collagen type II staining was dried for 30 min at 60 °C. Then samples were deparaffinized and endogenous peroxidase was blocked with 3% H_2_O_2_ (Thermo Fisher, Waltham, MA, USA) for 10 min and rinsed with distilled water. Slides were either steamed for 20 min in trisodium citrate buffer (pH 6.0) (ZUC028-500, Zytomed, Berlin, Germany) for collagen type I or treated with pepsin solution (Thermo Fisher, Waltham, MA, USA) for 10 min at 37 °C for collagen type II followed by rinsing for 5 min with tris-buffered saline (TBS). Samples were incubated in horse serum (S-2012, Vector Laboratories, Burlingame, CA, USA) for 20 min, incubated with the primary antibody (rabbit polyclonal anti-collagen 1 (Abcam, Cambridge, United Kingdom); mouse monoclonal anti-collagen 2 (MS-306-PO, Thermo Fisher, Waltham, MA, USA)) for 1 hour at room temperature (RT), rinsed with TBS for 5 min and incubated with the secondary antibody (collagen type I: goat anti-rabbit peroxidase labelled IG (Agilent Technologies, Santa Clara, CA, USA); collagen type II: goat anti-mouse peroxidase labelled IG (K4001, Agilent Technologies, Santa Clara, CA, USA)) for 30 min at RT, followed by rinsing with TBS for 5 min. For detection, slices were incubated for 3 min with 2–3 drops of VECTOR NovaRED (Vector Laboratories, Burlingame, CA, USA) and the reaction was stopped by submerging in water for 10 min. Afterwards, slides were counterstained with Hemalum (Roth, Karlsruhe, Germany) for 1 min and blued for 10 min in tap water. Slides were dehydrated, cleared with xylene (Roth, Karlsruhe, Germany) and covered with a glass slide.

### Determination of reactive oxygen species (ROS) via electron paramagnetic resonance (EPR) measurements

Differentiation medium was incubated with 500 µM of the spin probe cyclic hydroxylamine 1-hydroxy-3-carboxy-2,2,5,5-tetramethylpyrrolidine hydrochloride (CP-H; Noxygen, Elzach, Germany) for 4 hours under stimulated (4 bars, 2 s/2 s pattern) or under static condition. Additionally, to further clarify the underlying mechanisms, the iron chelator diethylenetriaminepentaacetic acid (DTPA), superoxide dismutase (SOD) or both DTPA and SOD were added to the medium. For analysis, medium was placed in 100 µL portions of 1 mL disposable pipettes (VWR International, Radnor, PA, USA) and snap frozen in liquid nitrogen. EPR spectra of frozen samples were recorded at 3359 ± 200 G using the Magnettech MiniScope MS 200 EPR spectrometer (Magnettech Ltd., Berlin, Germany)^25^. The general settings were as follows: modulation frequency 100 kHz, microwave frequency 9.425 GHz, microwave power 11 mW, modulation amplitude 7G. The magnitude of oxidized CP-H (3-CP) signals was calculated and is expressed in arbitrary units (AU).

### Quantitative reverse transcription polymerase chain reaction (RT-qPCR)

Cells were harvested on day 0, 21, and 42 by mincing the pellets with tissue grinding beads (Bertin corp., Rockville, MD, USA) in a tissue homogenizer (Precellys®24; Bertin corp., Rockville, MD, USA) followed by total mRNA extraction using the peqGOLD Total RNA Kit (VWR International, Radnor, PA, USA). RNA was measured using a NanoPhotometer (Implen GmbH, München, Germany) and 1 µg of mRNA was transcribed into cDNA using EasyScript™ cDNA Synthesis Kit (abm, Richmond, BC, Canada) using oligo(dT) primers. Quantitative PCR was performed using KAPA SYBR® FAST qPCR Kit (VWR International, Radnor, PA, USA) with a Stratagene© Mx3000P QPCR System (Agilent, Santa Clara, CA, USA) according to the manufacturer’s instructions using 10 ng of cDNA per reaction. Thermal cycle conditions were 5 min at 95 °C followed by 40 cycles of 10 s at 95 °C and 30 s at 60 °C (B2M, MMP3, RUNX2, IL6) or 30 s at 95 °C and 1 min at 60 °C (ACAN, COL1A1, COL2A1, COL10A1, SOX9, MMP9, MMP13, IL-1β, IL-6, TNF-α). For time-dependent expression profiles, target genes were normalized to the housekeeper β2-microglobulin (B2M) and compared to corresponding values of day 0 using the comparative CT (ΔΔCT) method. Primer sequences used are listed in Table 1.

**Table 1:** Primer sequences used for qPCR.

Target	Primer forward	Primer reverse
B2M (β2-Microglobulin)	GAT GAG TAT GCC TGC CGT GT	TGC GGC ATC TTC AAA CCT CC
ACAN (Aggrecan)	CCC CTG CTA TTT CAT CGA CCC	GAC ACA CGG CTC CAC TTG AT
COL1A1 (Collagen I)	GAT CTG CGT CTG CGA CAA C	GGC AGT TCT TGG TCT CGT CA
COL2A1 (Collagen II)	AGA CTT GCG TCT ACC CCA ATC	GCA GGC GTA GGA AGG TCA TC
COL10A1 (Collagen X)	CAT AAA AGG CCC ACT ACC CAA C	ACC TTG CTC TCC TCT TAC TGC
SOX9 (SRY-Box 9)	AGC GAA CGC ACA TCA AGA C	CTG TAG GCG ATC TGT TGG GG
MMP3 (Matrix Metallopeptidase 3)	ATG CCC ACT TTG ATG ATG ATG AAC	CCA CGC CTG AAG GAA GAG ATG
MMP9 (Matrix Metallopeptidase 9)	GTA CTC GAC CTG TAC CAG CG	TTC AGG GCG AGG ACC ATA GA
MMP13 (Matrix Metallopeptidase 13)	CCA GAC TTC ACG ATG GCA TTG	GGC ATC TCC TCC ATA ATT TGG C
RUNX2 (Runt Related Transcription Factor 2)	CCG TCT TCA CAA ATC CTC CCC	CCC GAG GTC CAT CTA CTG TAA C
IL-1β (Interleukin 1 β)	CAA CAG GCT GCT CTG GGA TT	GTC CTG GAA GGA GCA CTT CAT
IL-6 (Interleukin 6)	AGT TCC TGC AGA AAA AGG CAA AG	CAT TTG CCG AAG AGC CCT CA
TNF-α (Tumor Necrosis Factor α)	TCT CCT TCC TGA TCG TGG CA	GGG TTT GCT ACA ACA TGG GCT

### Quantification of matrix components

Biochemical assays were performed to quantify GAG and DNA content. Therefore, pellets were flash frozen in liquid nitrogen and digested with 500 µL proteinase K solution (≥30 units/mL proteinase K, 50 mM TRIS, 1 mM EDTA, 1 mM iodoacetamide, 10 µg/mL pepstatin A in ddH_2_O) at 56 °C overnight.

#### GAG quantification

GAG content of pellet cultures was determined using a dimethylmethylene blue (DMMB)-based staining assay. Briefly, 5 µL of the proteinase K-digested sample were diluted with 95 µL phosphate buffered EDTA (100 mM Na_2_HPO_4_ and 10 mM EDTA in PBS) in a flat bottom 96-well plate and a dilution series with chondroitin-4-sulfate in 1.75 mg/mL cysteine was made as standard. Both, 100 µL of diluted sample or standards were mixed with 200 µL DMMB solution (38.5 µM DMMB, 1% EtOH, 40.5 mM NaCl, 40.5 mM Glycine, and 9.5 mM Acetic Acid in ddH_2_O) and absorbance of samples was measured at 540 nm against 595 nm as reference wavelength using a plate reader (Sunrise Basic; Tecan Trading AG, Männedorf, Switzerland).

#### DNA quantification

DNA present in the pellets was quantified using the QuantiFluor® dsDNA kit (E2670; Promega, Madison, WI, USA). Briefly, 5 µL of proteinase K digested sample were diluted with 95 µL 1X tris-EDTA (TE) buffer in a black flat bottom 96-well plate and a standard curve was generated using provided Lambda DNA Standard. Sample and standards were mixed with 100 µL of 1X QuantiFluor® dsDNA dye and incubated for 5 min at room temperature (RT) in the dark before fluorescence measurements (Blue Fluorescence Optical Kit; 490 nm_Ex_/510–570 nm_Em_) using the GloMax®-Multi+ Detection Systems (Promega, Madison, WI, USA) were performed.

#### Viability assessment

Cell viability in pellets was assessed with a colorimetric assay using the standard methylthiazolyldiphenyl-tetrazolium bromide (MTT) method. Therefore, pellets were stimulated for 4 hours (4 bars, 2 s/2 s pattern) or cultured under static conditions. Pellets were transferred into a 48 well plate and incubated for 2 hours with 500 µL of MTT working solution (650 mg/mL MTT in ddH_2_O). MTT working solution was discarded and generated formazan was dissolved in 500 µl DMSO for 1 hour. Absorbance was measured at 540 nm wavelength against 650 nm as reference wavelength using a plate reader.

### Western Blot

PBS-washed pellets were flash frozen in liquid nitrogen and crushed into powder with tweezers. The powder was reconstituted in Nonidet P-40 buffer containing 40 mM HEPES (pH 7.9), 120 mM NaCl, 1 mM EDTA (pH 8.0), 10 mM 2-glycerolphosphate, 50 mM NaF, 0.5 mM Na_3_VSO_4_, 1% Nonidet P-40 substitute, and 1 mM Phenyl-Methyl-Sulfonyl Fluoride (PMSF) supplemented with 2 µg/mL aprotinin, 2 µg/mL leupeptin, 0.3 µg/mL benzamidine chloride, and 10 µg/mL trypsin inhibitor and lysed. Total protein of pellets was extracted by several freeze and thaw cycles. The protein extract was incubated on ice for 1 hour and centrifuged at 22,000 × g for 20 min at 4 °C. The supernatant of each sample was collected, transferred into a new vial and protein concentration was determined on a NanoPhotometer (Implen GmbH, Munich, Germany) using Bradford assay (Protein Assay Dye Reagent Concentrate; Bio-Rad, Hercules, CA, USA) according to manufacturer’s instructions. Equal amounts of protein (10 µg/lane) were applied to each lane on a SDS-polyacrylamide gel (10% running gel and 5% stacking gel) and run at increasing voltages (60, 80, 100 V). Then, the protein was transferred onto a nitrocellulose membrane (GE Healthcare, Little Chalfont, United Kingdom) and blocked with 5% nonfat milk powder in TBS buffer with 0.1% Tween (TBS/T). Primary antibodies were incubated at 4 °C overnight in 5% BSA in TBS/T and secondary antibodies were incubated at RT for 1 hour in 5% nonfat milk powder in TBS/T. Signals were detected using the Odyssey® Fc Imaging System (LI-COR, Lincoln, NE, USA) and assessed with Image Studio Lite (LI-COR, Lincoln, NE, USA) to generate ratios of phosphorylated protein to the total protein or housekeeper. Antibodies for phospho-AKT (Ser-473), total AKT, phospho-p44/42 MAPK (Thr-202/Tyr-204) (phospho-Erk1/2), total p44/42 MAPK (total ERK1/2), phospho-p38 MAPK (Thr-180/Tyr-182), total p38 MAPK, phospho-S6 ribosomal protein (Ser-240/244), total S6 ribosomal protein, β-Catenin, and GAPDH were obtained from Cell Signaling Technology (Danvers, MA, USA). The secondary antibodies IRDye® 680RD goat anti-mouse IgG, IRDye® 680LT donkey anti-rabbit IgG, IRDye® 800CW goat anti-mouse IgG, and IRDye® 800CW goat anti-rabbit IgG were obtained from LI-COR Biosciences (Lincoln, NE, USA).

### Statistical Analysis

Unless otherwise noted, all data are presented as mean + standard deviation (SD). All statistical calculations were performed using GraphPad Prism software (GraphPad Software Inc., San Diego, CA, USA). Normal distribution of values was tested using D’Agostino-Pearson omnibus test. Comparisons between two or multiple groups were calculated using Mann–Whitney *U* test or either one-way analysis of variance (ANOVA) with Tukey’s multiple comparison test or Kruskal–Wallis test with Dunn’s multiple comparison test, respectively. *P*-values < 0.05 were considered statistically significant.

## Results

### Generation of reactive oxygen species (ROS) via hydrostatic pressure (HP)

**Figure 3:**
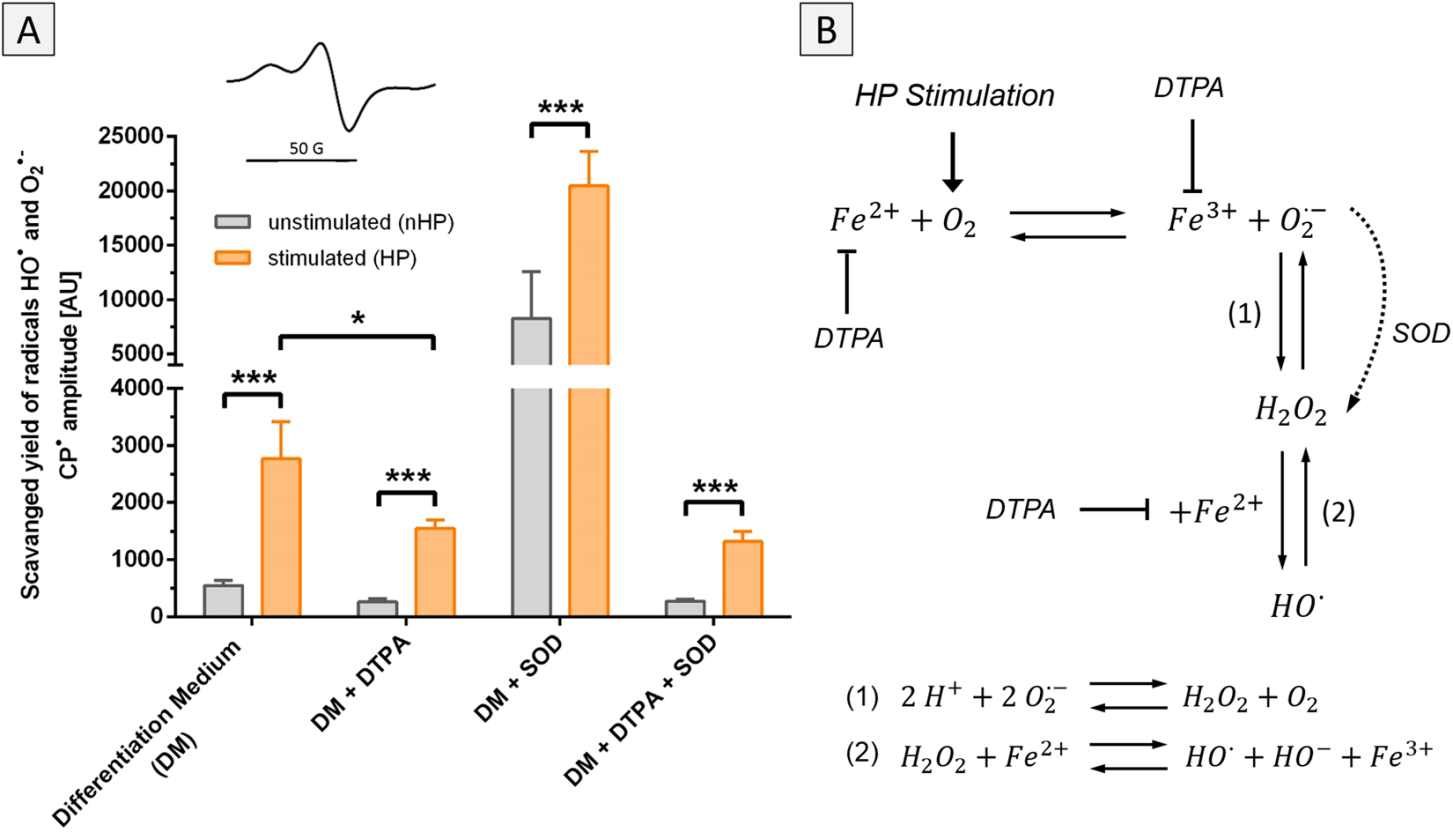
Acellular ROS formation by hydrostatic pressure stimulation. (**A**) DM without cells was stimulated according to the described pressure regime and generated reactive oxygen species (ROS) were measured using the spin probe CPH. Pressurized DM showed a five times higher amount of ROS than unpressurized medium. Adding DTPA into the medium to capture free iron (Fe^2+^/Fe^3+^) led to a substantial reduction of ROS (DM + DTPA). In contrast, the addition of SOD led to an enhanced accumulation of ROS (DM + SOD). The pressurized group showed almost twice the amount of ROS in the stimulated medium compared to unstimulated medium. Adding both SOD and DTPA simultaneously (DM + DTPA + SOD) hindered the accumulation of ROS and resulted in ROS levels comparable to the group where just DTPA was added. Inset: EPR signal of 3-CP. (**B**) Hydrostatic pressure stimulation increases partial oxygen pressure in the medium. In combination with free iron (Fe^2+^), superoxide (O_2_^•−^) is generated which is further converted via SOD into hydrogen peroxide (H_2_O_2_). H_2_O_2_ can then interact with free iron (Fe^2+^) to generate hydroxyl radicals (HO^•^). Using DTPA to bind free iron reduces production of superoxide and therefore also generation of other ROS. *p < 0.05, ***p < 0.001.

In a series of EPR-experiments, ROS-formation as a result of HP stimulation was verified and the involved reactive oxygen species and mechanisms were elucidated. Stimulation of DM resulted in approximately five times higher levels of ROS compared to unstimulated control medium (Fig. 3A). DTPA was added to show the involvement of iron ions in the radical generation process. The addition of the iron-chelator led to significantly lower ROS generation, whereas the addition of SOD resulted in high levels of ROS. SOD converted superoxide to hydrogen peroxide (H_2_O_2_), which is then further converted to hydroxyl radicals (HO^•^). The conversion of SOD was driving the main reaction of Fe^2+^ and O_2_ to Fe^3+^ and superoxide, which resulted in high ROS levels (Fig. 3B). SOD and DTPA simultaneously added to the differentiation medium hindered the accumulation of ROS, as the detected levels were similar to DTPA addition alone.

### HP stimulation prevents cartilage matrix formation

**Figure 4:**
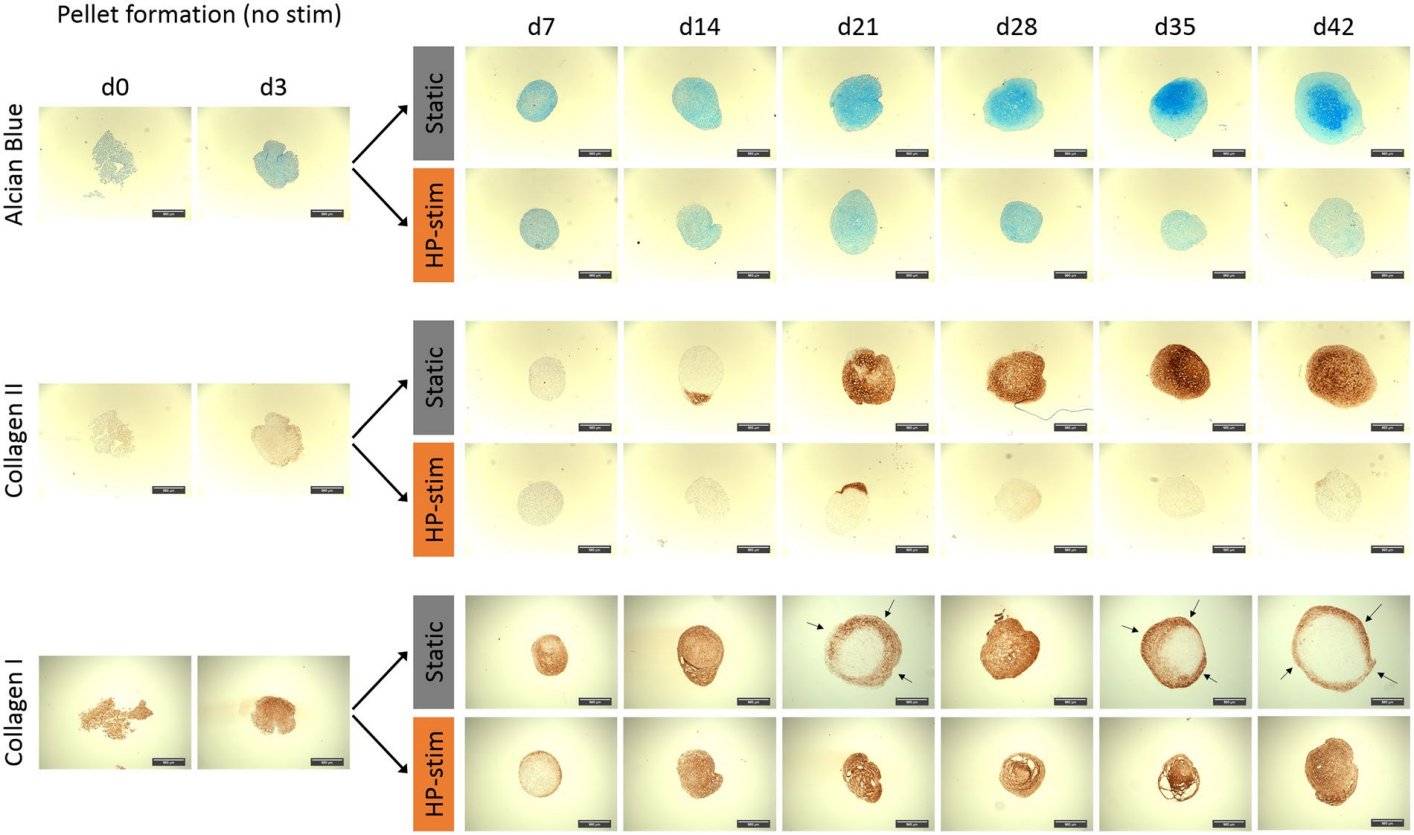
Histological analysis of chondrogenic ASC-pellet cultures under static and hydrostatic pressure stimulated (HP-stim) conditions. Static chondrogenic cultured pellets show an increasing amount of glycosaminoglycan (determined via alcian blue staining) and collagen II (determined via immunohistochemical staining) over time. In contrast, HP-stimulated pellets are negative for these markers but show uniform distribution of collagen I during the full culture time of 42 days. Static pellets also express collagen I, especially at earlier time points, but generally at the outer region of the pellets (indicated by black arrows). Scale bar: 100 µm.

The influence of elevated ROS levels on cartilage matrix formation in pellet cultures was investigated by histological analysis of pellets cultured for up to 42 days. Three days after pellet formation, one group of pellets was stimulated with HP according to the described pressure regime (Fig. 2B), whereas an unstimulated group of pellets served as control. These unstimulated pellets showed increased positive stainings for both cartilage-specific extracellular matrix (ECM) components collagen II and GAGs, stained via immunohistochemistry and alcian blue staining, respectively (Fig. 4). In contrast to unstimulated pellets, HP-treated pellets showed nearly no formation of collagen II and only low expression of GAGs. In both groups, collagen I was substantially expressed; in the stimulated group generally throughout the whole pellet, whereas in the unstimulated control group the staining was limited to the outer region of the pellet.

### HP stimulation degrades preformed cartilage matrix

**Figure 5:**
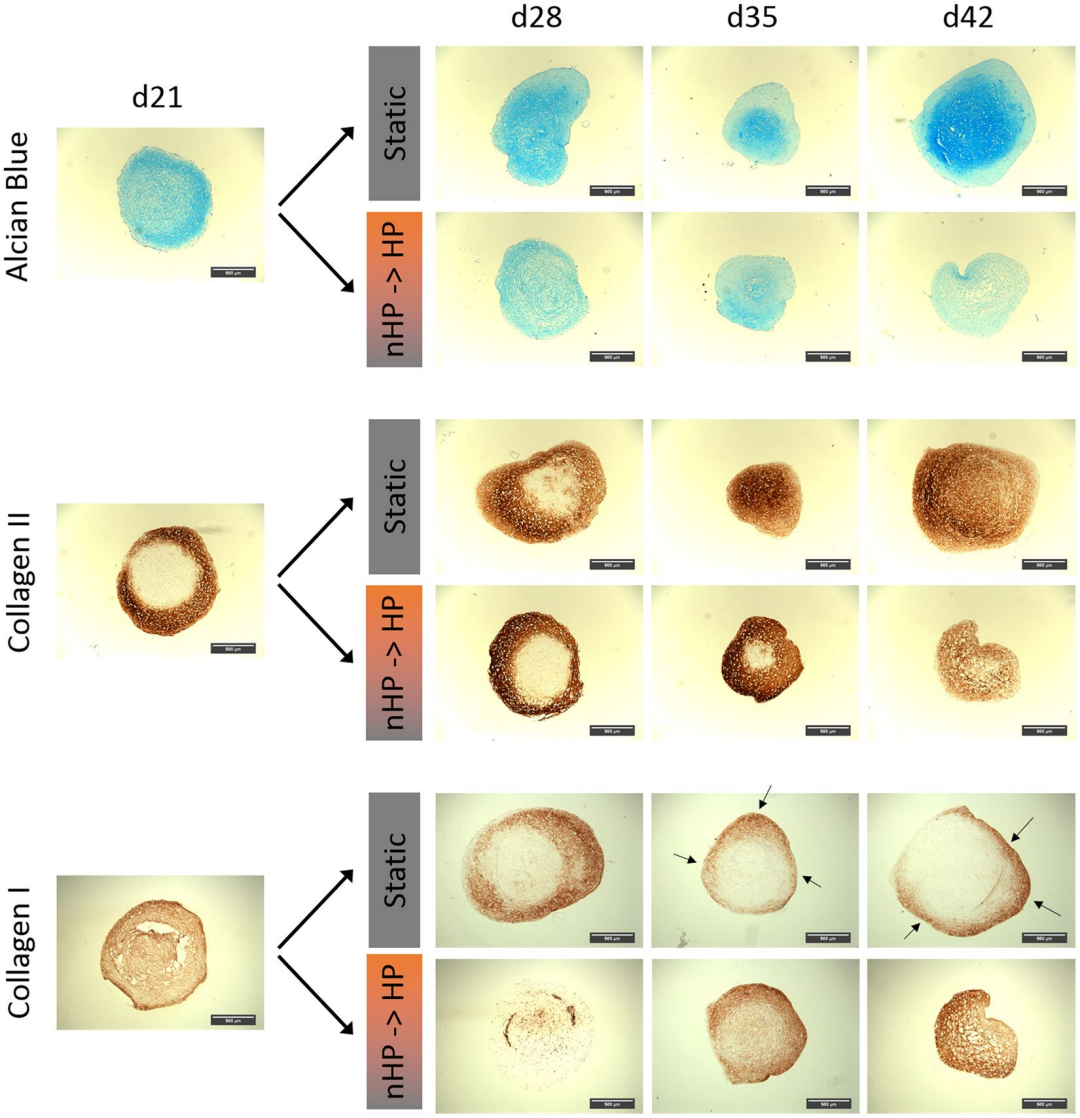
Histological analysis of chondrogenic ASC-pellet cultures under static and switched hydrostatic pressure (nHP → HP) stimulated conditions. Until day 21, pellets of both groups were cultured under static conditions and formed substantial amounts of GAGs (determined via alcian blue staining) and collagen types I and II (determined via immunohistochemistry stainings). Afterwards, static pellet cultures gradually increased GAG content as well as collagen II content whereas collagen I content decreased gradually and was only locally expressed in the outer shell of the pellet structures (indicated by black arrows). Pellets cultured under switched conditions steadily lost pro-chondrogenic markers (alcian blue, collagen II) after day 21 but showed an increased collagen I content throughout the whole pellet. Scale bar: 100 µm.

After observing the inhibitory effect of HP stimulation on cartilage formation in pellet cultures, the focus was to determine whether the HP stimulation also leads to degradation of cartilage matrix. Therefore, unstimulated static pellet cultures were cultured for 21 days in which a significant amount of GAGs and collagen II was formed. Pellets then received HP stimulation for another 21 days (Fig. 2A). As a control, pellets were cultured for a total of 42 days without stimulation under static conditions. After 21 days of static culture, positive staining for GAGs and collagen II was observed (Fig. 5), which further increased under static conditions but decreased in stimulated groups over time. HP stimulation led to a uniform collagen I expression throughout the pellets, whereas in static controls only the margins of the pellets were positively stained.

**Figure 6:**
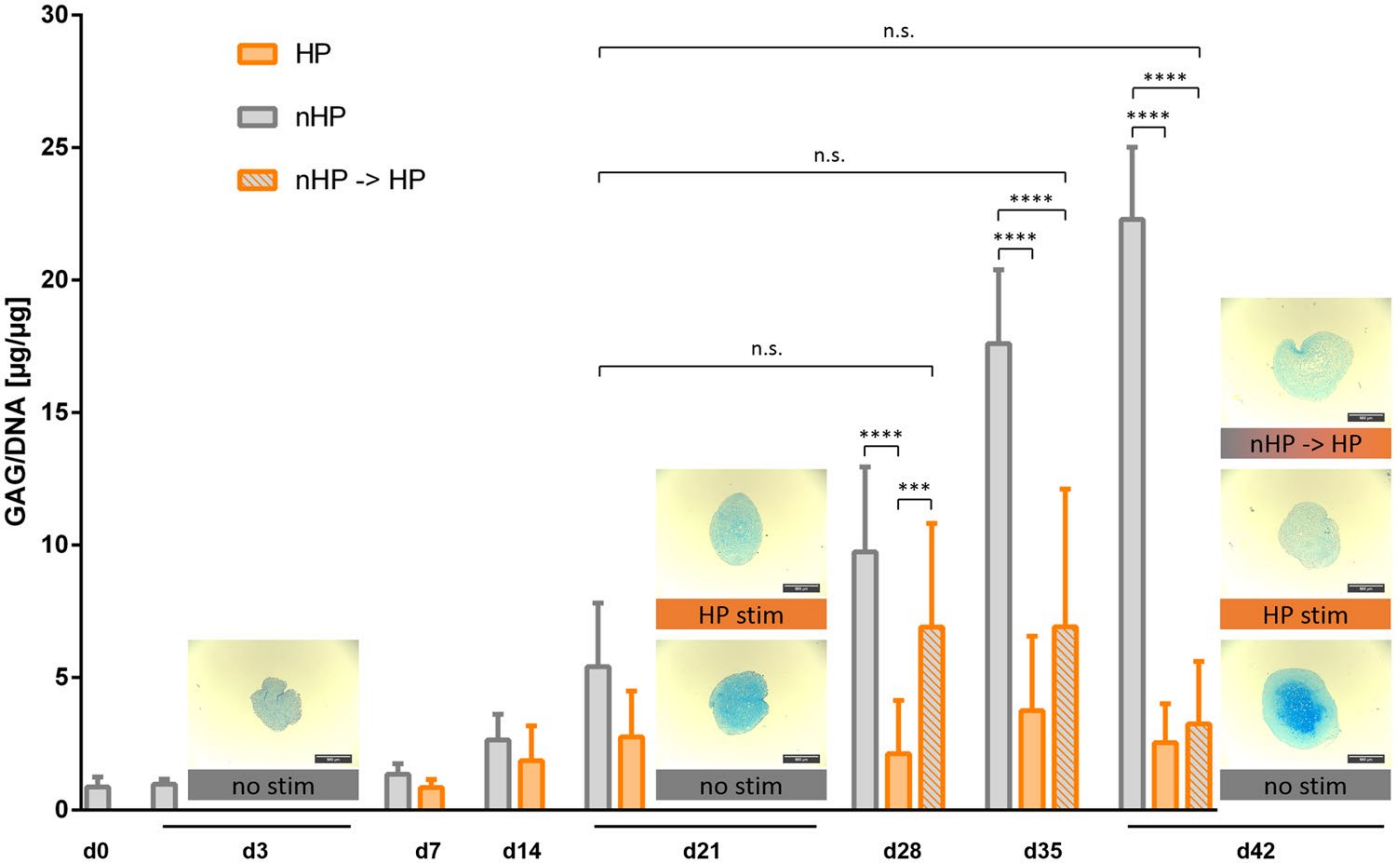
Influence of hydrostatic pressure stimulation on quantitative glycosaminoglycan (GAG) to DNA amount. Static cultured pellets increased GAG to DNA ratio gradually over a 6 weeks culture period and expressed a 10-fold difference to continuously HP-stimulated pellets and a 7-fold difference to switched stimulated pellets on day 42, respectively. On day 21, continuously stimulated pellets showed a reduction of produced GAG compared to unstimulated pellets. Switched stimulated pellets that were cultured under static conditions for 21 days and then experienced hydrostatic loading for another 21 days marginally increased GAG/DNA ratio on day 28 and day 35 but had a GAG to DNA ratio on day 42 similar to pellets cultured continuously with HP for 42 days. Representative examples of alcian blue stained pellets next to the respective bars qualitatively underline the results from the quantitative GAG to DNA ratio. Data from 3 individual donors, 4 replicates per donor; **p < 0.01, ****p < 0.0001.

Quantitative GAG to DNA content measurements confirmed the histological staining analysis that HP stimulation affects GAG content in a dual manner: (1) pellets stimulated from day 3 on showed inhibited GAG deposition compared to unstimulated static controls and (2) pellets under switched conditions showed a decreasing amount of GAG content indicating degradation of already formed ECM matrix (Fig. 6, Supplementary Fig. S1A). Although the GAG/DNA amount of pellets stimulated after day 21 increased marginally from day 28 to day 35, the final ratio (3.2 µg GAG/µg DNA) on day 42 was below the original value (5.4 µg GAG/µg DNA) of static cultured pellets on day 21. Pellets stimulated continuously for 42 days showed a significant difference of produced GAG already on day 21 compared to unstimulated pellets, gradually increasing to a 10-fold difference on day 42. Similar to continuously stimulated pellets, pellets that were stimulated after day 21 (after a pre-chondrogenic differentiation phase) showed a significant 7-fold difference on day 42 compared to static culture pellets.

### HP stimulation decreases viability of pellets over time

Besides the inhibitory and degenerative effect on cartilage formation, especially on GAGs (Supplementary Fig. S1B), pellets subjected to HP stimulation displayed a reduced amount of DNA (Supplementary Fig. S1C). DNA amount was highest on day 0 but decreased over one week to stabilize and remain on the same level for the rest of the experiment for static cultured pellets. In contrast, DNA amount of continuously stimulated pellets gradually decreased from day 7 to less than 50% of the starting value on day 42. Similarly, DNA of switched stimulated pellets dropped progressively from day 28 and reached their lowest value on day 42. Furthermore, to increase validity of short-term DNA data, viability of pellets was checked after one day of stimulation as well as one week of stimulation (Supplementary Fig. S1D). One day of HP stimulation did not lead to any adverse effect on stimulated pellets. Similarly, one week of stimulation did not have a significant effect on the pellets either, which is in accordance with DNA data of day 7 (Supplementary Fig. S1C).

### RT-qPCR

#### Hydrostatic pressure (HP) decreases collagen type II to I ratio

**Figure 7:**
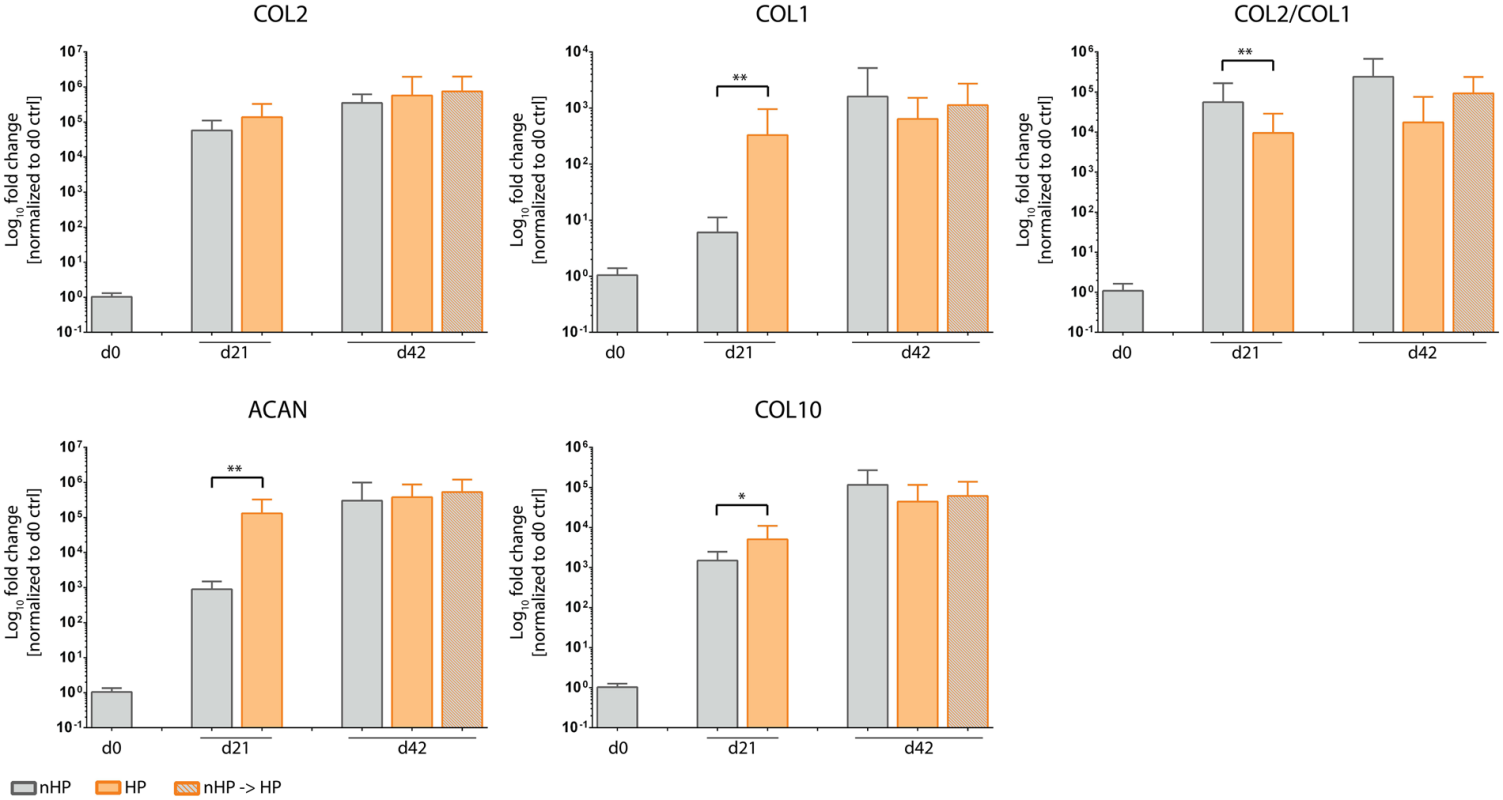
Influence of hydrostatic pressure (HP) stimulation on gene expression of extracellular matrix proteins. The cartilage differentiation index, a ratio of collagen type II to collagen type I (Col2/Col1), was significantly downregulated on day 21 for HP-stimulated compared to static cultured pellets but showed no difference on day 42. Collagen type II of both stimulated groups was expressed at levels equal to the control group on day 21 as well as on day 42. Expression of collagen type I, aggrecan and collagen type X was significantly upregulated in HP-stimulated groups on day 21 but was equally expressed on day 42 for all groups. Data from 3 individual donors, 5 replicates per donor; *p < 0.05, **p < 0.01.

Transcription levels of cartilage-specific genes were tracked using RT-qPCR. The ratio of collagen type II to collagen type I mRNA levels (COL2/COL1), commonly used as a cartilage differentiation index^26^, was significantly lower only in stimulated pellets after 21 days of culture, whereas no significant difference between static and any HP-stimulated group could be detected on day 42 (Fig. 7). Investigating the levels of collagen type II and collagen type I mRNA separately revealed that collagen type I mRNA expression is stronger influenced by HP stimulation than collagen type II. Cartilage-specific collagen type II expression was comparable between static and stimulated groups on day 21, as well as on day 42 for all groups. Collagen type I levels of the stimulated groups were significantly upregulated on day 21 but showed no difference compared to other groups on day 42. In contrast to histological stainings, expression of aggrecan, a proteoglycan and major structural component of articular cartilage, was significantly upregulated on day 21 in stimulated pellets but was equally expressed on day 42 in all groups. Similar to aggrecan, collagen type X, which is an early marker for hypertrophic chondrocytes, was significantly upregulated on day 21 but was not impacted in either of the HP-stimulated groups on day 42 compared to control.

#### HP stimulation upregulates expression of transcription factors SOX9 and RUNX2

**Figure 8:**
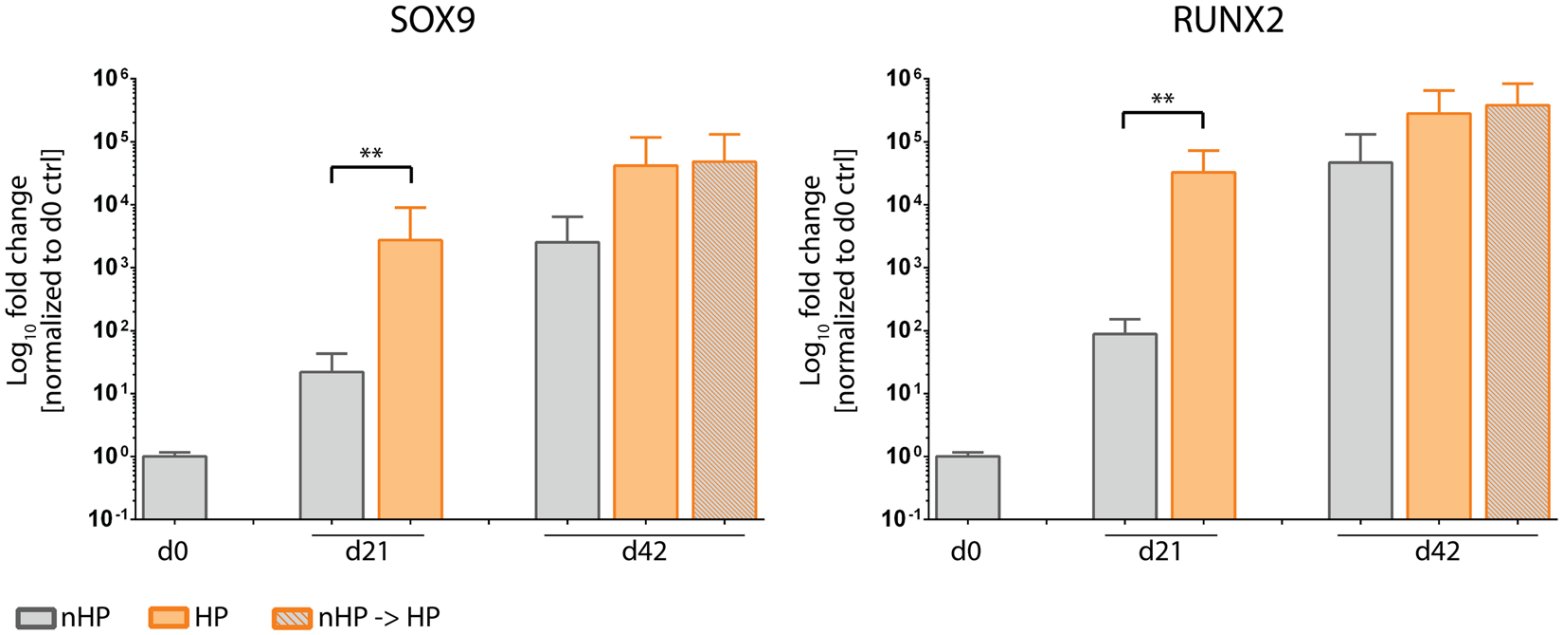
Influence of hydrostatic pressure (HP) stimulation on gene expression of transcription factors. Both investigated transcription factors SOX9 and RUNX2 were significantly upregulated in HP-stimulated pellet cultures compared to static pellet cultures on day 21. Although not significantly different, HP-stimulated pellets had higher levels of SOX9 as well as RUNX2 on day 42. Data from 3 individual donors, 5 replicates per donor; **p < 0.01.

Expression of SOX9, a crucial transcription factor for chondrocytes, was significantly upregulated after HP stimulation compared to control on day 21 but equally expressed in all groups on day 42 (Fig. 8). Similarly, RUNX2, a key osteoblastic transcription factor, was significantly upregulated in stimulated pellets on day 21 but equally expressed among all groups on day 42.

#### HP-stimulated pellet cultures show augmented expression of matrix metalloproteinases (MMPs)

**Figure 9:**
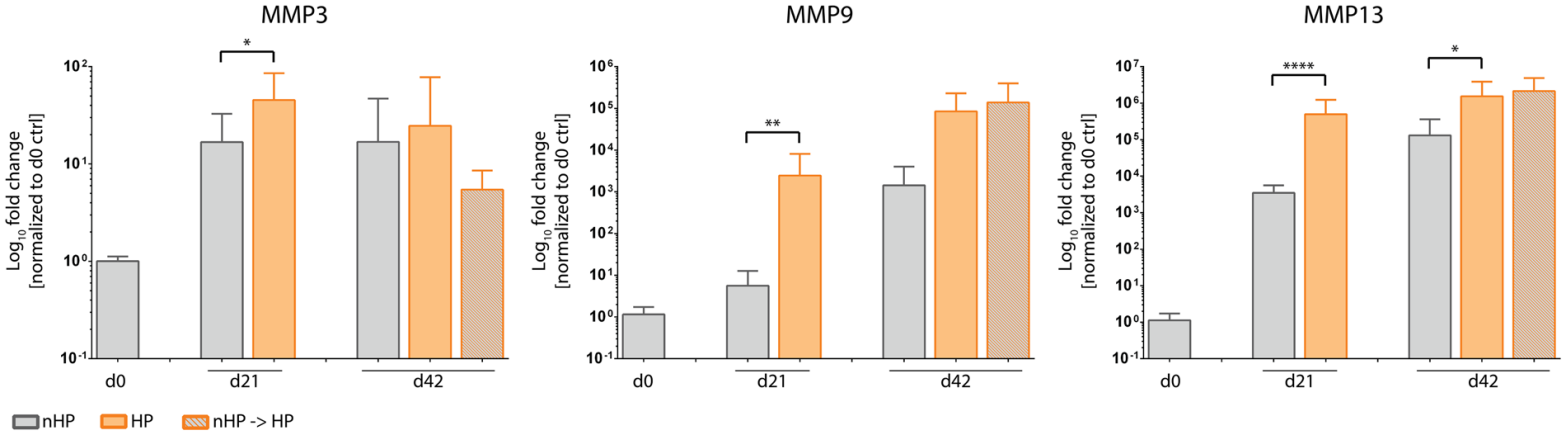
Hydrostatic pressure (HP) stimulation increases gene expression of matrix metalloproteinases proteins. Gene expression of all investigated MMPs (MMP3, MMP9 and MMP13) was significantly upregulated in hydrostatic stimulated pellets on day 21. MMP9 and MMP13 had higher expression in both HP-stimulated groups on day 42 with MMP13 being significantly different to the continuously stimulated group. Data from 3 individual donors, 5 replicates per donor; *p < 0.05, **p < 0.01, ****p < 0.0001.

After observing a loss of GAGs and collagen type II in HP-treated pellets (Fig. 4), the expression of matrix metalloproteinases – essential in cartilage remodelling and osteoarthritis – was investigated. MMP3 (e.g. known to degrade cartilage proteoglycans), MMP9 (e.g. known to degrade different types of collagen) and MMP13 (e.g. known to cleave collagen type II) are described as the main mediators of cartilage matrix degradation in overstimulated and/or osteoarthritic cartilage^27,28^. On day 21, the expression of all three MMPs was significantly upregulated following HP stimulation in comparison to unstimulated control pellets (Fig. 9). On day 42, expression levels for MMP3 and MMP9 were not significantly different between stimulated and static cultured pellets. Only MMP13 expression was enhanced for both HP-stimulated pellet groups compared to static controls, being significantly upregulated in HP-stimulated pellets from day 3 on. No difference was observed between the two HP stimulation regimes.

#### Upregulation of OA-associated pro-inflammatory cytokines by HP stimulation

**Figure 10:**
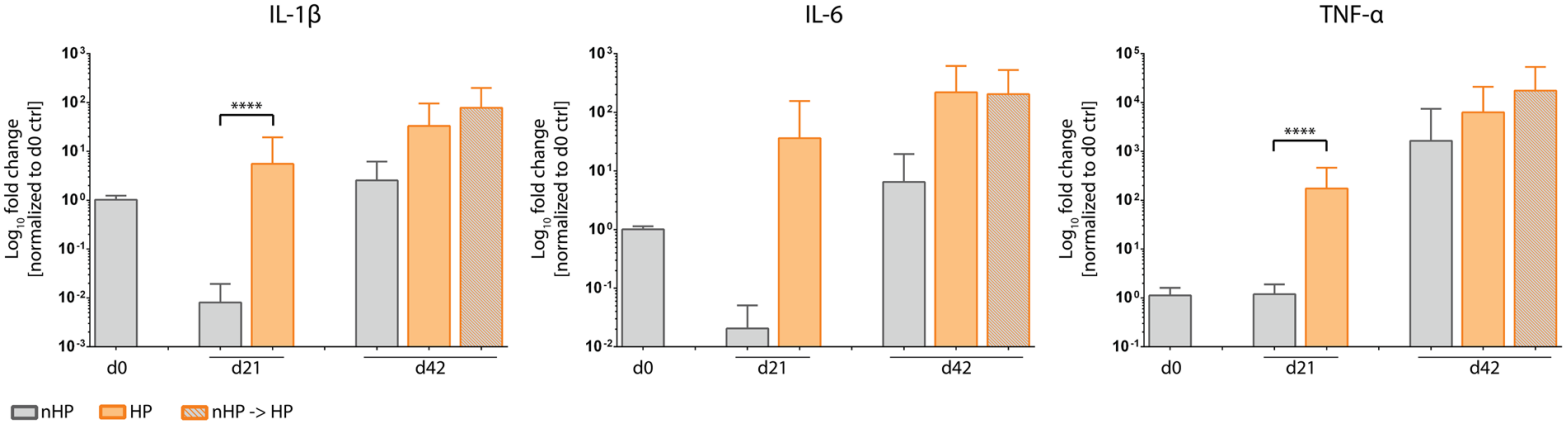
Hydrostatic pressure (HP)-stimulated pellet cultures show increased gene expression levels of inflammatory cytokines. In comparison to the static group, HP-stimulated pellet cultures showed increased gene expression of the pro-inflammatory cytokines IL-1β, IL-6 and TNF-α on day 21. On day 42, in both HP-stimulated groups IL-1β, IL-6 and TNF-α expression was upregulated compared to unstimulated pellets. IL-6 was equally highly expressed in both HP-stimulated groups. Compared to day 0, unstimulated pellets harvested on day 21 had reduced IL-1β and IL-6 gene expression, though TNF-α was similarly expressed. Data from 3 individual donors, 5 replicates per donor; ****p < 0.0001.

In order to investigate the effect of HP stimulation on the expression of inflammation-related cytokines, three different pro-inflammatory cytokines which are generally upregulated in OA – IL-1β, IL-6 and TNF-α – have been investigated (Fig. 10). On day 21, HP-stimulated pellets demonstrated increased expression levels of all three cytokines compared to static controls, with IL-1β and TNF-α being significantly different (p < 0.0001). On day 42, a difference between all three pellet conditions could be observed and showed a trend towards elevated levels in HP-stimulated groups compared to static control pellets for all 3 investigated cytokines. Interestingly, expression of IL-1β and IL-6 in control pellets was lower on day 21 than on day 0.

### Western Blot

**Figure 11:**
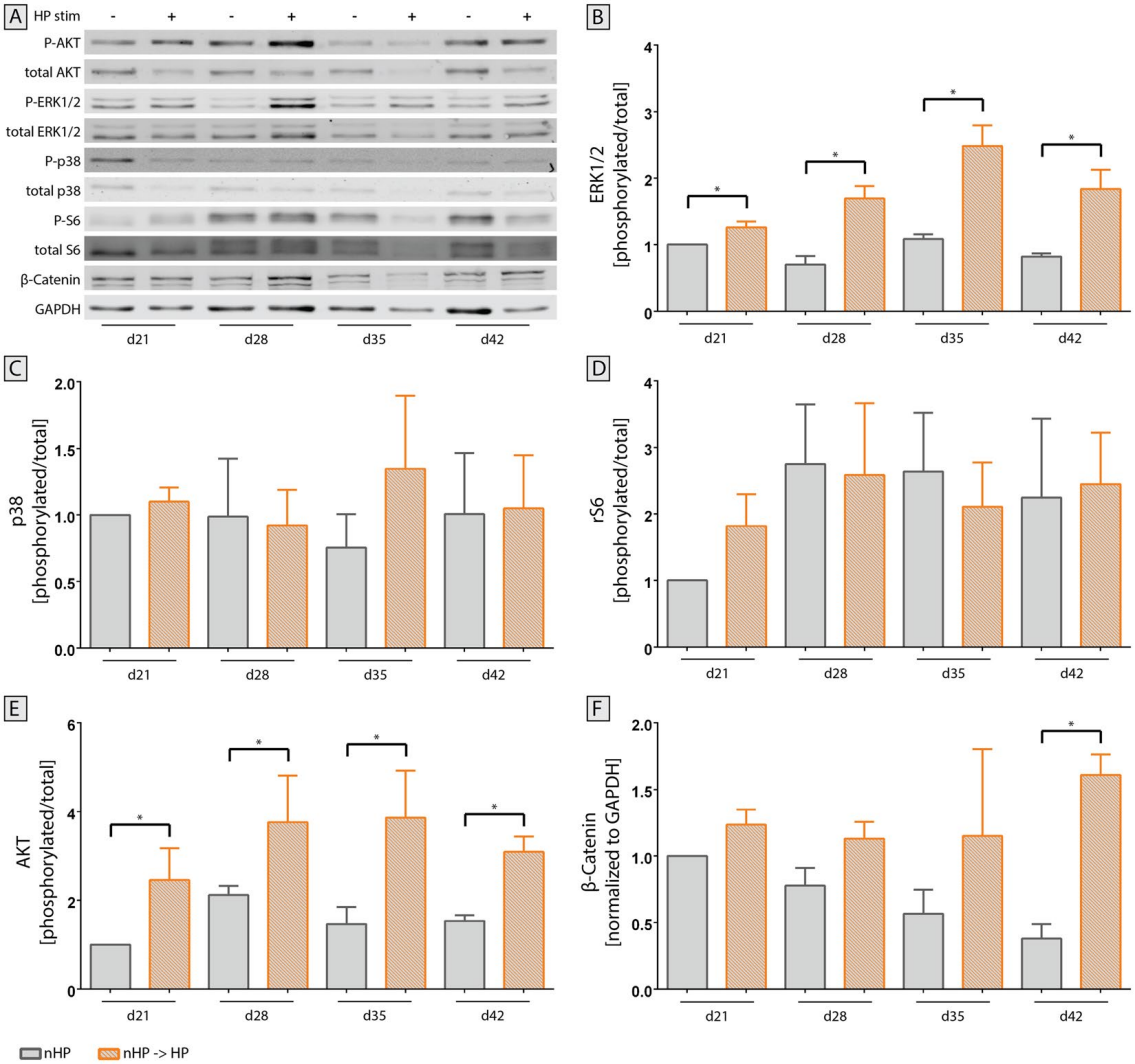
Activation of crucial signaling pathways in osteoarthritis after hydrostatic pressure (HP) stimulation. (**A**) Representative immunoblots from the same gel were cropped to show specific bands for phosphorylated and total protein of Akt, ERK1/2, p38, ribosomal protein S6 as well as β-Catenin using GAPDH as housekeeping protein. Full-length blots are presented in Supplementary Figure S2. Pellets of unstimulated and HP-stimulated groups were harvested once every week for the last 21 days of the experiment and protein was isolated. Both (**B**) ERK1/2 and (**E**) Akt showed a statistically significant increase in activation in HP-stimulated pellets compared to static controls at all timepoints. (**C**) Protein expression of p38 did not significantly change over time. (**D**) Ribosomal protein S6, a downstream target of ERK1/2 and mTOR, did not exhibit an enhanced activation at any sampling timepoint. (**F**) Expression of β-Catenin increased towards the end of the stimulation period for stimulated pellets whilst it declined for unstimulated pellets. Mean + SEM; data from 2 individual donors, 6 replicates for each donor; *p < 0.05.

Subsequently to gene expression analysis, prominent signaling targets of upregulated inflammatory cytokines – ERK1/2 and p38 MAPK – were investigated on the protein level in the switched stimulated pellet group (nHP → HP) compared to unstimulated static pellets. Analysis of immunoblots of proteins related to OA (Fig. 11A) showed that, starting with day 21, stimulated pellets showed higher ERK1/2 activation compared to unstimulated static pellet cultures (Fig. 11B). This trend continued over time and reached its maximum on day 35 but declined at the end of the stimulation period (day 42 of pellet culture). In contrast, p38 MAPK did not follow such a pattern with comparable early activation levels of stimulated and static pellets (Fig. 11C). This was also the case for ribosomal protein S6 (Fig. 11D), which is involved in the regulation of cell size and cell proliferation. Furthermore, Akt, a major component in the canonical mTOR pathway and downstream target of inflammatory cytokines, showed an activation profile similar to ERK1/2. Compared to unstimulated pellets, HP-stimulated pellets exhibited a higher level of activation for Akt at each time point over the entire experimental period (Fig. 11E). To investigate another possible trigger of OA, β-Catenin was investigated. Compared to unstimulated pellets on day 21, stimulated pellets showed an increased expression pattern towards the end of the stimulation period, whilst the expression in unstimulated pellets gradually decreased (Fig. 11F).

## Discussion

Osteoarthritis (OA) represents a burden for a growing number of people across the globe, especially due to the increased prevalence in risk factors leading to OA, such as obesity and a sedentary lifestyle^29^. Despite intensive research over the past 20 years, a detailed understanding of the triggers and mechanisms leading to initiation and progression of this degenerative joint disease is still incomplete^30^. Historically, OA was described as a simple “wear-and-tear” type disease but is now accepted to be a more complex disease in which inflammation processes play a critical role^3^. In recently published studies, elevated ROS levels due to oxidative stress are associated with formation and progression of cartilage degradation as seen in OA^6,31,32^.

In the presented study, the application of hydrostatic pressure (HP) via a custom-made bioreactor system (Fig. 1) led to generation of acellular ROS. Via a series of EPR measurements we could demonstrate that superoxide (O_2_^•−^) is initially generated, which subsequently reacts to produce other ROS (Fig. 3) including hydrogen peroxide (H_2_O_2_) and hydroxyl radicals (HO•). Notably, the increased HP leads to generation of elevated levels of acellular ROS. Interestingly, elevated levels of HP have also been described in OA-affected joints^33,34^. In knee joints, these elevated intra-articular fluid pressure levels are attributed to effusions which occur in over 80% of patients^35,36^. Another cause for elevated intra-articular fluid pressure is body weight, a well-known OA risk factor. In this regard, Felson *et al*. describe that gaining 10 pounds body weight results in approximately 30 pounds more load on the knee during walking^37^. In general, excessive joint loadings, either a single acute impact event or repetitive cumulative contact stresses, are regarded as main contributor in the pathogenesis of OA^38,39^. Via *in vitro* studies using bioreactor systems^17,40,41^ to mimic joint loading it could be demonstrated that excessive mechanical stimulation of articular cartilage initiates the production of ROS and reactive nitrogen species. This oxidative stress is then the primary trigger for the characteristic inflammation process associated with OA.

Additionally, EPR measurements indicated that free iron is a critical component of acellular ROS formation after HP stimulation. Notably, elevated synovial iron levels have been indicated in patients with degenerative joint diseases such as rheumatoid arthritis and OA^42,43^. The origin of iron is suggested to be blood which enters the joint due to trauma or secretion from inflamed areas of synovial membranes^42^. Joint bleeding leads to iron release from haemoglobin which induces an inflammatory environment mediated by cytokines and hydroxyl radical formation^44,45^. Non-protein-bound iron has been investigated as a trigger but also as a marker for degenerative joint diseases^46^. In this regard, Kawai *et al*.^47^ showed that IL-1-treated rats developed arthritis which was accompanied by statistically higher free iron levels in synovial fluid compared to saline-treated controls.

There is consensus that elevated levels of reactive oxygen and nitrogen species directly damage chondrocytes, for example by lipid peroxidation^48^ or DNA damage^49^ and lead to disturbed collagen type II and GAG synthesis as well as to enhanced expression of matrix metalloproteinases (MMPs)^50–52^. Moreover, ROS, especially hydrogen peroxide, are described to fragment link proteins and to inhibit association of proteoglycan monomers with other ECM components (e.g. hyaluronic acid)^53,54^. The above described dual effect could also be seen in the presented study. HP stimulation, and thereby generated ROS, led to a reduction of already formed GAG and collagen type II (Fig. 5) as well as to an inhibition of the formation of these cartilage-specific ECM proteins in chondrogenic MSC pellets (Fig. 4). The degradative effects occurred despite the culture media containing potent chondrogenic growth factors TGF-β3 and BMP6. We could clearly indicate that the HP stimulation significantly upregulated the expression of all three investigated MMPs 3, 9 and 13 (Fig. 9). This was further accompanied by a severe loss in cartilage matrix proteins in chondrogenically pre-differentiated pellets over time. One limitation of this study is that it is still to be investigated if the formation of cartilage matrix was hindered directly by disturbing essential chondrogenic signaling pathways, by the expression of matrix-degrading MMPs or as a result of both effects. Another factor that most likely additionally affected the reduced GAG content of the stimulated pellets is the induction of apoptosis due to the HP-induced oxidative stress. HP-treated pellets showed significantly lower amounts of DNA which can be directly correlated to reduced cell numbers. Despite the possible induction of apoptosis, no direct effects on cell viability could be detected (Supplementary Fig. S1). Hence, more specific experiments need to be executed in future studies to address these questions and decipher the detailed mechanisms.

In contrast to collagen type II and GAG downregulation, collagen type I expression was upregulated, which could be seen in RT-qPCR (Fig. 7) as well as in the histological analyses (Figs. 4 and 5). The upregulation of collagen type I additionally contributed to a decrease in collagen type II to collagen type I ratio, which has been used as a differentiation marker for healthy cartilage cells^26,44,55^. Remarkably, the exposition to ROS in the experimental set-up was not accompanied by collagen type III expression (data not shown), as it has been described for injured regions of articular cartilage by Hosseininia *et al*.^56^, comparable to wound healing and scar tissue formation in skin or tendon^57,58^.

Gene expression analysis in our study further included the OA-associated cytokines IL-1β, TNF-α and IL-6. It is well reported that upregulation of cytokine expression can be linked to increased ROS levels and can play an important role in the pathogenesis of OA. For instance, Davies *et al*. demonstrated that IL-1β mediates ROS-induced DNA damage in osteoarthritic cartilage^59^. Interestingly, IL-1β and IL-6 expression in control pellets on day 21 was reduced compared to day 0 levels (Fig. 10), which might have been caused by mechanical stress during the pelleting procedure. Furthermore, the catabolic effect of these interleukins on articular cartilage has been associated with activation of different signaling pathways including MAPK/ERK and PI3K/Akt. Different studies reported the ERK pathway as a negative regulator of chondrogenesis. For instance, Wang *et al*. showed that IL-1β enhances MMP3 and MMP13 expression but inhibits collagen type II and aggrecan via simultaneous MAPK/ERK pathway activation^60^. In this regard, Mio *et al*. have shown that ERK pathway activation leads to suppression of SOX9 expression in hydrostatic pressure-treated chondrocytes^61^. Mechanical loading-induced ERK1/2 phosphorylation leads to a decrease in proteoglycan synthesis in cartilage explant cultures^62^.

Different signaling pathways including ERK1/2 and Akt have been investigated in unstimulated static cultures compared to pellets pre-differentiated into the chondrogenic lineage for 21 days in which OA-like conditions have then been induced (nHP → HP, Fig. 11). In accordance with literature^63^, upregulation of OA-associated gene and protein expression in the presented study could be associated with increased ERK1/2 activation. Similar to MAPK/ERK, also the PI3K/Akt signaling pathway was activated via HP stimulation treatment. The activation of the PI3K/Akt pathway can result in diverse regulations due to its broad range of target proteins such as mTOR, NF-κB, GSK-3β, and p53^64^. Nevertheless, PI3K/Akt has been reported to be involved in OA regulation and progression since its overactivation leads to inflammation and apoptosis of chondrocytes^65,66^. Activation of Akt and thereby induced apoptosis could be linked to the observed reduced pellet size and DNA content in HP-stimulated group compared to unstimulated samples (Figs 4 and 5, Supplementary Fig. S1). Interestingly, Lopez-Armada *et al*.^67^ have proposed that apoptosis can lead to increased ROS production which might facilitate chondrocyte death.

Besides Akt and ERK1/2, other targets including p38 MAPK and β-Catenin were activated but less pronounced; activation of Akt and ERK1/2 was up to 4-fold higher, whereas the activation of p38 MAPK and β-Catenin was only 1.5-fold higher relative to unstimulated controls on day 21. In this regard Cheleschi *et al*. could show that cyclic HP can lower the β-Catenin expression in OA chondrocytes which might therefore be the key modulator for the restored expression of miRNAs dysregulated in OA leading to significant reduction of proteases including MMP13. It is likely that the influence of the described bioreactor system on these signaling pathways, previously demonstrated to be involved in OA initiation and progression^68,69^, can be statistically verified through repetition of the experiment.

Unlike other signaling molecules, ribosomal S6 protein did not appear to be activated by HP stimulation. This is in contrast to other studies which showed that the severity of OA could be reduced in preclinical and animal models^70^ by both pharmacological and genetic deletion of mTOR.

This study mainly describes the effects of the observed acellular ROS formation due to HP treatment on ASC chondrogenic pellets but did not investigate the direct effects of mechanical deformation on the cellular integrity. In literature, HP treatment is described to lead to ultrastructural and cytoskeletal changes of 2D cultured OA-chondrocytes^11,13^. Future studies are needed to elucidate if the applied HP similarly leads to changes of the cytoskeleton of individual cells cultured in pellet cultures.

## Conclusion/Outlook

In summary, a custom-made hydrostatic pressure bioreactor system capable of generating acellular ROS was utilized to generate an *in vitro* model for OA that produced comparable biological effects known for initiation and progression of the degenerative joint disease. In future studies, the created system will be used to test antioxidant properties of numerous drugs proposed as potential OA treatments^71^, such as aucubin^17^ or curcumin^72^. Another possible application may pursue the involvement of free iron and oxygen stress-related signaling pathways present in OA in order to elucidate possible novel targets for future therapeutic approaches.

## Electronic supplementary material

Supplementary Information
