# Supplementary material for: Hydrostatic pressure-generated reactive oxygen species induce osteoarthritic conditions in cartilage pellet cultures
Source: Sci Rep. 2018 Nov 19;8:17010. doi: 10.1038/s41598-018-34718-8 (PMC6242959; doi:10.1038/s41598-018-34718-8)
Supplement: Supplementary file 1 — Supplementary Information [file 41598_2018_34718_MOESM1_ESM.pdf]

Hydrostatic pressure-generated reactive oxygen species induce osteoarthritic conditions in cartilage pellet cultures

Bernhard Rieder[1], Anna M. Weihs[1], Adelheid Weidinger[2], Dorota Szwarc[1], Sylvia Nürnberger[3], Heinz Redl[2], Dominik Rünzler[1], Carina Huber-Gries[1], Andreas H. Teuschl[1,*]

[1]Department Life Science Engineering, University of Applied Sciences Technikum Wien, 1200 Vienna, Austria

[2]Ludwig Boltzmann Institute for Experimental and Clinical Traumatology, AUVA Research Center, 1200 Vienna, Austria

[3]Department of Orthopedics and Trauma-Surgery, School of Dentistry Medical University of Vienna, 1090 Vienna, Austria

*corresponding author: teuschl@technikum-wien.at

Supplementary Figure S1:

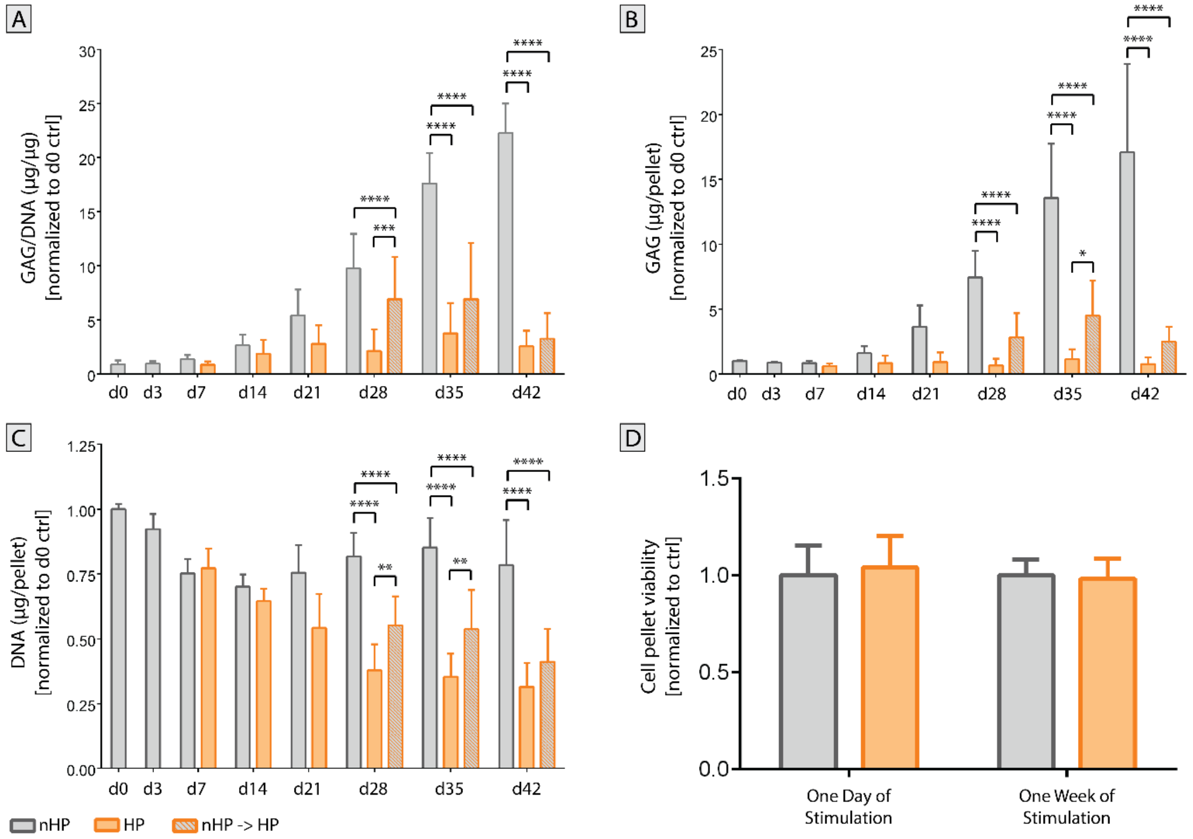

Supplementary Fig. S1: Influence of hydrostatic pressure (HP) stimulation on viability of cells. (A) One day of stimulation did not adversely affect stimulated cells. Similarly, one week of stimulation did not have a significant effect on the pellets either. **(B)** GAG to DNA ratio gradually increased over the 6 weeks period for the static cultured pellets and expressed a 10-fold difference compared to continuously HP-stimulated pellets, as well as a 7-fold difference to switched stimulated pellets on the last day of the experiment. **(C)** Complementary to the GAG/DNA ratio, the amount of GAG did increase for the static cultured pellets but stayed on the same level for the continuously HP-stimulated pellets over the course of the experiment. Pellets that were stimulated after day 21 till the end of the experiment expressed the least amount of GAG on day 42. **(D)** Amount of DNA in unstimulated pellets was highest on day 0, decreased for one week but stabilized on day 7 for the rest of the experiment. In contrast, DNA content of continuously HP-stimulated pellets did gradually decrease over time with the lowest DNA value on day 42. Similarly, DNA amount of switched stimulated pellets dropped progressively from values similar to static control to values comparable to continuously HP-stimulated pellets at the end of the experiment. (A) Data from 2 individual donors, 10 replicates per donor (B,C,D) Data from 3 individual donors, 4 replicates per donor, **p < 0.05, **p < 0.01, ***p < 0.001, ****p < 0.0001

Supplementary Figure S2:

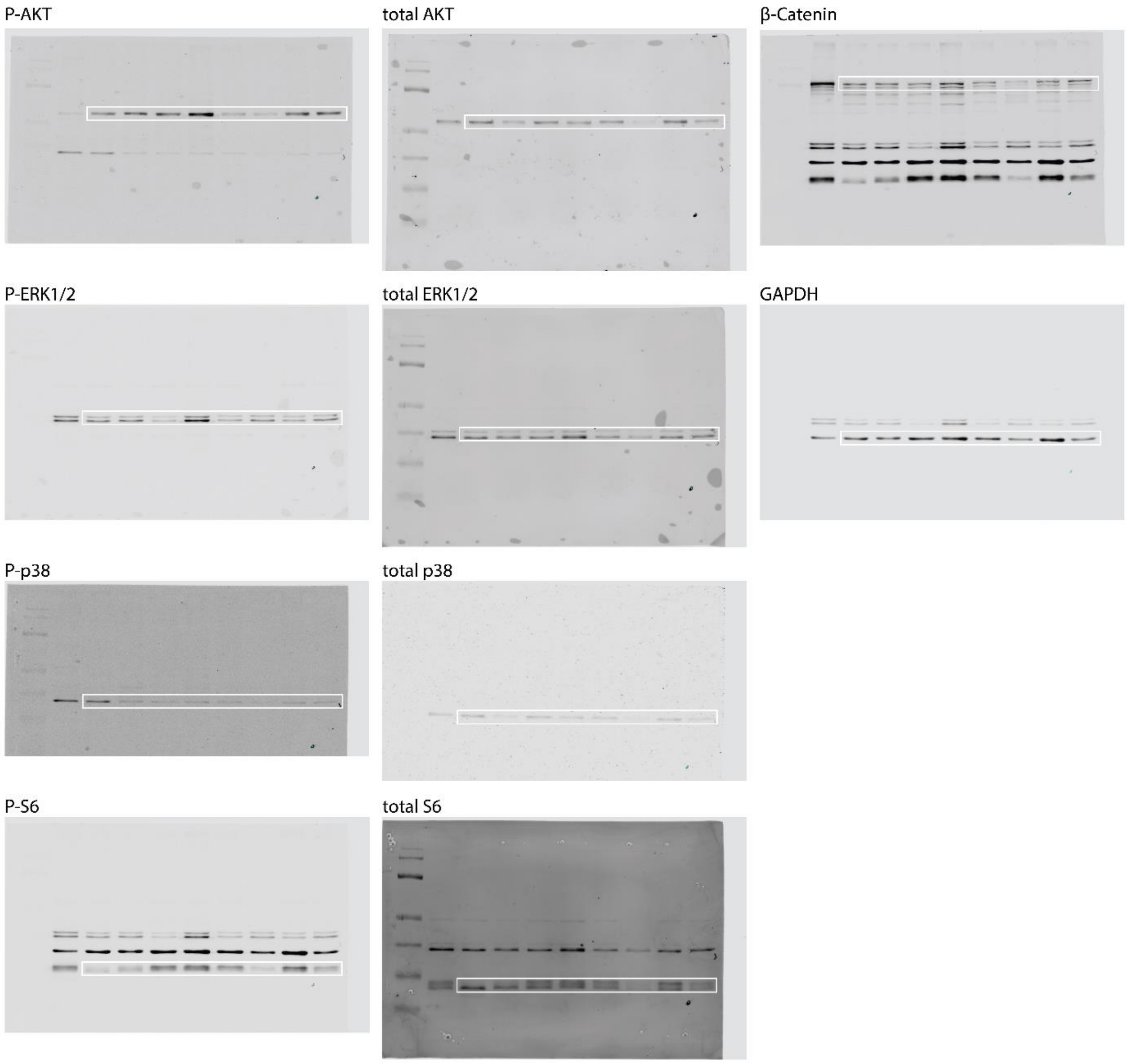

Supplementary Fig. S2: Full-length blots of P-Akt, total AKT, phospho-p44/42 MAPK (phospho-Erk1/2), total p44/42 MAPK (total ERK1/2), phospho-p38 MAPK, total p38 MAPK, phospho-S6 ribosomal protein, total S6 ribosomal protein, β-Catenin, and GAPDH. Areas marked with a white rectangle were used for Figure 11A.
